# Indents

**Published:** 1919-11

**Authors:** 


### INDENTS

**☞Brief Articles of Technical or Scientific Value will receive notice and comment. Contributions invited.**

---

Supports for Work in Porcelain Furnace. It is not always possible to buy the desired supports for our crown and inlay work, owing to market prices, but most of us can obtain a piece of soapstone from a plumbing shop and from this cut any desired support. After the first heating it is almost impossible to cut the block.—F. W. Frahm, in *Pacific Gazette.*

---

Relation of Teeth to the Ridge in a Lower Denture. By setting the lower teeth directly over the lower ridge, and as close to the ridge as esthetics will permit, the stability of the lower denture is greatly increased. Taken bucco or labio-lingually, the center of any given lower tooth should be directly above the middle line of the lower ridge. When the lower teeth are placed in this manner the tongue is allowed ample room, and the buccal and labial muscles are prevented from exerting a dislodging pressure on them in either speech or mastication. The objection may be raised here that upper teeth will be set an unnecessary distance outside of the ridge, due to the fact that the lower ridge is usually considerably larger than the upper. This is so, but the area of the upper affords greater possibilities for retention, and the upper denture rests on an immovable supporting member which more than offsets any increased leverage caused by setting the upper teeth outside of the ridge when this is necessary. —Russell W. Tench, in *Dental Digest.*

---

The Oral Hygienist. Some twenty years ago, Dr. Frank W. Low, of Buffalo, had a vision which he embodied in a paper read before one of our local societies in Buffalo. The title of the paper was ''The Odontocure,'' and the essayist predicted that the operation of oral prophylaxis in the future would be performed by one who had special training in this class of work. I do not know where my friend Dr. Low got his inspiration, but gentlemen, the "odontocure" is here, and I believe has come to stay, as she is vouched for by this society and has the government seal upon her. The necessities of the situation demand her presence. An oral hygiene propaganda extending over a considerable number of years has created a condition that can be met in no other way. The broad application of the underlying principles of oral prophylaxis has opened up a wide field and placed new responsibilities upon the profession, and the dentist who ignores their relative importance in his daily practice has not a proper conception of this important branch of dentistry.—Dr. R. Murray, in *Cosmos*.

---

Metal Antagonizing Casts. It is very desirable, if not a necessity, to have the antagonizing teeth in partial dentures and crown and bridge work made of metal. Accuracy in every detail is demanded by the careful operator. The final results will more than repay the small expense and additional time that are required. Make an accurate modeling compound or plaster impression. Amalgamate and soften about a half ounce of copper amalgam and pack the occlusal half and cutting edges of all the teeth in the impression, without an attempt at smoothing off the amalgam surface; then run the remainder of the cast in the usual manner and separate after the case has stood for twelve hours. The amalgam can be used a number of times providing the mercury balance has not been disturbed.—F. W. Frahm, in *Pacific Gazette*.

---

Dental Reconstruction and Bolshevism. The Editor has received a letter in which the writer commends in the following words Dr. Stengle's article upon the subject of "The Relation of Dental Affections to Systemic Disease," which appeared in July *Cosmos* and August *Oral*

*Health*: "So far as I have gone into Dr. Stengle's article, I find it much saner than most medical doctrines that are being taught today." Dr. Stengle warns the dental profession that "we hear a great deal of loose talk about the teeth and the desirability of getting rid of them."

The dental profession in these days of wonderful reconstruction requires the steadying influence of conservatism. In the matter of the extraction of teeth, the pendulum has, in many localities, swung entirely too far in the direction of ruthless extraction.

Reconstruction is popular. All good citizens are determined that a new order shall prevail, and are energetically discarding the old and building up the new, to the end that truth, liberty and justice may obtain throughout the wide world. And this same commendable spirit is abroad in dentistry. Scientific recognition that septic conditions in and about the teeth lead to serious systemic disease, has forced a marked reconstruction of the principles and practices of dentistry. One might almost say there has occurred a dental revolution, so far-reaching have been the changes that have taken place in the practice of dentistry during the past half decade.

This is all very good, but unfortunately "Bolshevism" has crept in, whose disciples of destruction see nothing but sepsis, disease and death in every pulpless tooth or gingival inflammation. A policy of indiscriminate extraction has resulted, with the consequent sacrifice of sound, healthy teeth, the marring of natural beauty, loss of comfort and serious depreciation in masticatory and digestive efficiency.

Unfortunately there are dentists who blindy follow the advice of medical practitioners, without making careful examination and dependent diagnosis. They are *consenting parties* to these outrages, *without giving thoughtful and intelligent consent.*

Let us not forget that, notwithstanding the criticisms that are so frequently hurled at the dental practices of yesterday, and in spite of the many failures and mistakes of the past, dentistry has rendered in the aggregate an inestimable service to humanity and to public health during the past half century. We have occasion to feel proud of dentistry's record and the many advances she has made. made.

It was recently reported in the press that the death of that illustrious world citizen, Theodore Roosevelt, was directly due to an infected tooth. It is such cases as this that serve in a striking though tragic way to bring home to the public mind the desirability and absolute necessity of maintaining the mouth and teeth in a condition of cleanliness and health. It is, however, the duty of the dentist to take nothing for granted, but make a careful study and diagnosis of each case, and advise extraction only when such treatment is indicated. No dentist, whether he be general practitioner or exodontic specialist, should in the practice of his profession receive instructions from a medical practitioner regarding diagnosis and treatment. Cooperation and consultation are essential, but, in the final analysis, it is the manifest duty of the dental practitioner to make his own diagnosis of the dental conditions present and then outline a conservative treatment best suited to the case.—Editorial in September *Oral Health.*

